# Semi-Targeted Nuclear Magnetic Resonance Metabolomics via Parahydrogen-Induced Hyperpolarization for Enhanced Sensitivity to Metabolic Composition

**DOI:** 10.1021/jacs.5c11226

**Published:** 2025-08-26

**Authors:** Thom B. Posthumus, Udo F. H. Engelke, Ruud L. E. G. Aspers, Jona Merx, Thomas J. Boltje, Jonathan Martens, Ron A. Wevers, Martin C. Feiters, Floris P. J. T. Rutjes, Marco Tessari

**Affiliations:** † Institute for Molecules and Materials, Radboud University, Heyendaalseweg 135, 6525 AJ Nijmegen, The Netherlands; ‡ Translational Metabolic Laboratory, Department Human Genetics, Radboud University Medical Centre, Geert Grooteplein Zuid 10, 6525 GA Nijmegen, The Netherlands

## Abstract

Non-hydrogenative para-hydrogen-induced polarization (nhPHIP) has proven a powerful tool for the enhanced NMR detection of several classes of metabolites in complex mixtures. Particularly, compounds carrying an α-amino acid motif have been previously detected and quantified in biological samples and natural extracts at submicromolar concentrations using 2D nhPHIP NMR spectroscopy. This technique is here applied for the first time in a semi-targeted metabolomics NMR study on urine from patients suffering from Pyridoxine-Dependent Epilepsy (PDE), currently diagnosed by the presence of dilute unique biomarkers. The signal enhancement, combined with the resolving power of 2D nhPHIP, results in a superior sensitivity to the metabolic composition compared to conventional ^1^H NMR, demonstrating its potential in NMR-based metabolomics.

## Graphical abstract

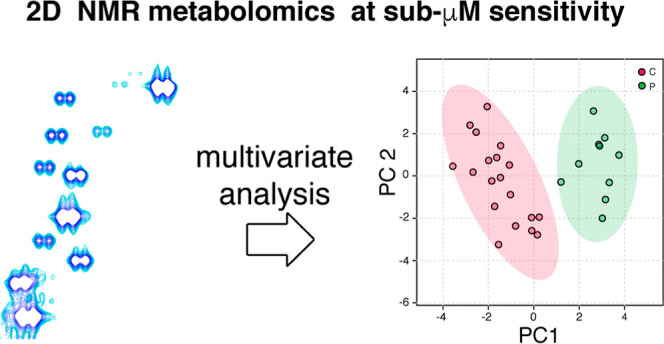

## Introduction

Over the last decades, NMR has gained an important role in metabolomics, for a large part due to its wide analyte scope, the minimal sample preparation, its reproducibility, and unique quantitative character.^1–4^ NMR-based metabolomics rely mostly on 1D ^1^H measurements, which afford sensitivity and wide applicability, thanks to the ubiquitous presence of proton spins in bioorganic molecules. However, analytes at low- or submicromolar concentrations remain generally below the NMR detection limit, particularly when dealing with complex biological samples where the number of components and the reduced ^1^H spectral window further hamper the measurement of more dilute species.

Different approaches based on hyperpolarization techniques have been proposed for the NMR detection of compounds at low concentrations. These methods realize transient conditions in which nuclear spin polarization is increased by orders of magnitude with respect to thermal equilibrium, resulting in large NMR signal enhancements. One such technique, dissolution Dynamic Nuclear Polarization (*d*-DNP), has recently been applied to an NMR clinical study on urine, with promising results in the metabolite coverage, thanks to the resolving power of hyperpolarized ^13^C NMR spectra measured at a natural abundance.^5^

Alternatively, an NMR signal enhancement can be obtained for different classes of compounds (e.g., nitrogen- and sulfur-containing heteroaromatic compounds, nitriles, amines, Schiff bases, diazirines, α-amino acids, and oligopeptides) via non-hydrogenative parahydrogen-induced hyperpolarization (nhPHIP).^6–15^ The selective character of nhPHIP-NMR makes it suitable for semi-targeted metabolomics to access low concentrations of the aforementioned classes of metabolites.^16^ While this selectivity might appear as a limitation in the context of metabolomics, the resulting spectral simplification can aid in highlighting biomarkers that are not detectable by conventional NMR due to the spectral crowding and/or excessive dilution. This is well illustrated in the present metabolomic study where, for the first time, a nhPHIP semi-targeted analysis was implemented on urine samples from patients with pyridoxine-dependent epilepsy (PDE-ALDH7A1). PDE, an inherited metabolic disorder, derives from mutations in the gene encoding α-aminoadipic semialdehyde (α-AASA) dehydrogenase, also known as antiquitin.^17^ Antiquitin is involved in lysine (**1**) catabolism in the brain and liver and, if mutated, causes accumulation of several intermediate metabolites, including pipecolic acid (**4**), α-AASA (**2**), Δ^1^-piperideine-6-carboxylic acid (Δ^1^-P6C, **5**), and 6-oxopipecolic acid (**6**).^18,19^ Unique biomarkers (**7–10**, Figure 1) have been reported in urine, which form from the reactive equilibrium α-AASA/Δ^1^-P6C.^20–22^

**Figure 1:**
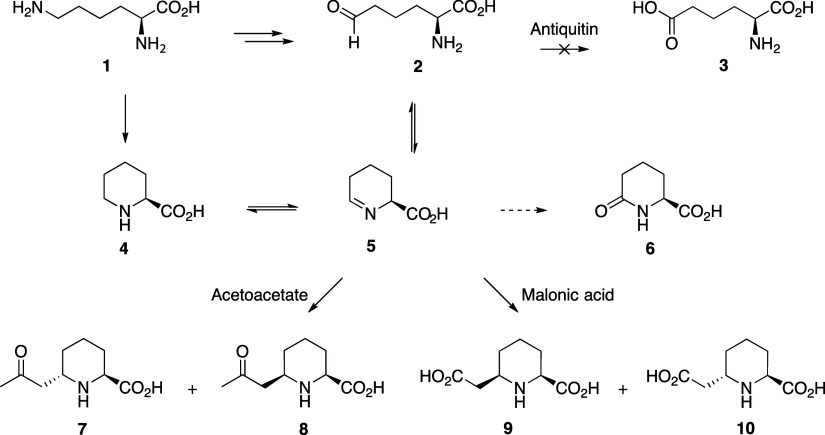
Overview of the lysine catabolism pathway, including biomarkers (**6**–**10**) formed in PDE patients. Numbered compounds: **1**: lysine, **2**: α-aminoadipic semialdehyde (a-AASA), **3**: aminoadipic acid, **4**: pipecolic acid, **5**: Δ1-piperideine-6-carboxylic acid (Δ1-P6C), **6**: 6-oxopipecolic acid, **7**: (2*S*,6*S*)-6-(2-oxopropyl)­piperidine-2-carboxylic acid, **8**: (2*S*,6*R*)-6-(2-oxopropyl)­piperidine-2-carboxylic acid, **9**: (2*S*,6*R*)-6-(carboxymethyl)­piperidine-2-carboxylic acid, and **10**: (2*S*,6*S*)-6-(carboxymethyl)­piperidine-2-carboxylic acid. Compounds **2** and **4**–**10** are known PDE biomarkers.

Because all these metabolites carry an α-amino acidic group, they are detectable via nhPHIP-NMR.^12^ Our semi-targeted nhPHIP-NMR metabolomics study indeed indicates a superior sensitivity to metabolic composition compared to a conventional ^1^H NMR approach, providing a clear distinction between the PDE and the control groups. The increase in the NMR signal and the resolution offered by the proposed method allowed the detection and identification of several biomarkers responsible for the observed group separation.

## Theory

nhPHIP is based on the reversible association of a substrate molecule to a complex of iridium, together with para-enriched hydrogen (*p*-H_2_).^6,16^ The sketch in Figure 2A displays this transient complex when a generic α-amino acid associates to the catalyst, with *p*-H_2_ and pyridine as coligands.^12^

**Figure 2:**
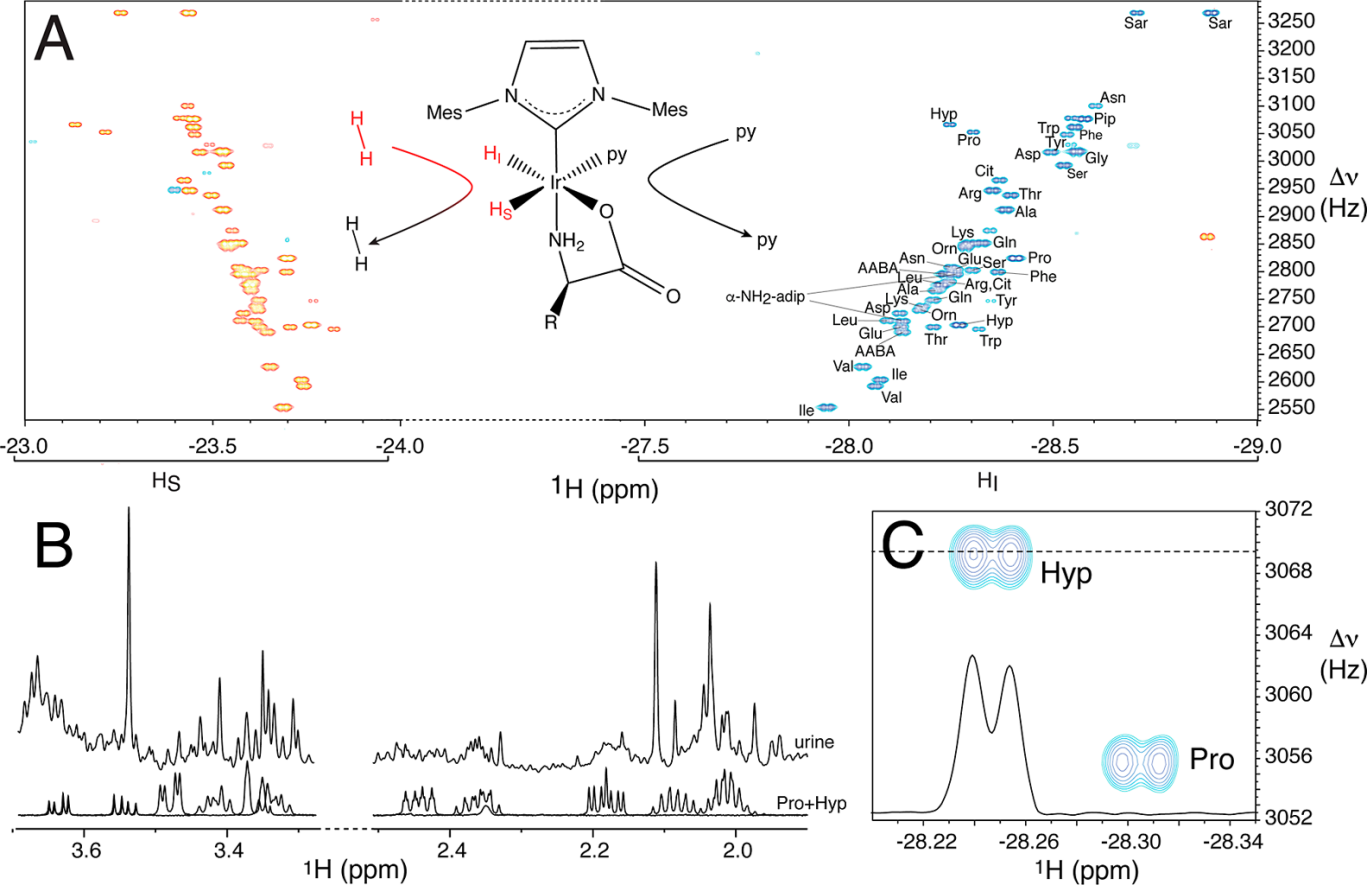
(A) 2D nhPHIP zero quantum (ZQ) hydride spectrum of a mixture of 34 α-amino acids (10 μM concentration each) in MeOH/H_2_O 95:5 (v/v). The spectrum was acquired at 10 **°**C in 1 h at 600 MHz ^1^H resonance frequency, in the presence of 0.43 mM Ir-IMes catalyst, 7.3 mM pyridine as cosubstrate, 10.2 mM piperidine buffer at pH 11.1, and 5 bar 51% *p*-H_2_. IMes stands for 1,3-bis­(2,4,6-trimethylphenyl)­imidazole-2-ylidene. Amino acid assignment is indicated for the H_I_ hydrides. The structure of the transient complex formed upon the association of an α-amino acid, *p-*H_2_, and pyridine to the Ir-IMes catalyst is sketched. The red font for H_2_ in solution indicates singlet order, while the black font indicates thermal hydrogen. The red font for the dihydride indicates longitudinal spin order. (B) Selected regions of conventional ^1^H urine spectra containing the aliphatic signals of l-proline and l-hydroxyproline for a urine sample (top) and a mixture of the two amino acids (bottom). (C) Portion of the 2D nhPHIP ZQ hydride spectrum measured on the same urine sample used for (B) (after 20-fold dilution) displaying the signals of l-proline and l-hydroxyproline. l-hydroxyproline is traced to illustrate the signal-to-noise ratio.

Because of the asymmetry in the equatorial plane, the singlet order originating from *p*-H_2_ can be converted at a high magnetic field into hydride magnetization, which is detected via NMR with up to 1000-fold enhanced sensitivity.^15^ Sample repolarization can be achieved by bubbling *p*-H_2_ through the solution at the beginning of each transient, allowing signal averaging as well as the acquisition of multidimensional NMR spectra at a submicromolar concentration.^23,24^

For each α-amino acid complex, a pair of hydride resonances at well-defined chemical shifts is observed.^12^ These hyperpolarized hydrides can, therefore, be used as chemosensors to indirectly reveal the presence of specific amino acids in solution. When dealing with complex mixtures, such as biological samples, the overlap of the hydride resonances can be resolved by 2D zero quantum (ZQ) spectroscopy: hydride signals corresponding to different α-amino acids can be spread in the indirect dimension, according to their zero-quantum frequency (i.e., the frequency difference within the dihydride),^25^ as shown in Figure 2A.

A major benefit of this chemosensing approach is the fact that hydrides resonate well below −20 ppm, in a region of the ^1^H spectrum that does not overlap with the background signals originating from the sample matrix.^24^ Therefore, nhPHIP-NMR measurements can be directly performed on the intact sample (after a simple dilution in methanol) without any prior fractionation. Note that dilution in methanol is an important requirement when working on aqueous solutions, such as urine, to minimize the negative effects of water on the nhPHIP efficiency. Furthermore, urine dilution prevents saturating the catalyst with α-amino acids, which is important for extracting quantitative information from nhPHIP data.^12^

The removal of the spectral background from the sample matrix, together with the enhanced sensitivity and resolution of 2D nhPHIP-NMR can provide direct access to dilute metabolites that are usually hard to detect because of signal overlap. This is exemplified in Figure 2B, illustrating how the detection of the aliphatic signals of l-proline and l-hydroxyproline can be greatly hampered in conventional ^1^H NMR spectra of urine because of signal crowding. Conversely, their measurement by 2D nhPHIP-NMR is quite straightforward, as displayed in Figure 2C for the same urine sample.

## Results

Among all biofluids, urine is certainly the most complex one, with several thousand predicted metabolites, spanning a concentration range of at least 11 orders of magnitude.^26^ Other than e.g. blood plasma, where metabolite levels are tightly regulated by homeostasis, urine displays considerable sample-to-sample differences in composition and concentration.^26,27^ These typical features pose additional complications in urine metabolomics, such as noninduced biological variations and heteroscedasticity. All in all, urine represents a challenging system to test the performance of nhPHIP hyperpolarization applied to NMR metabolomics.

In the present work, 1D and 2D nhPHIP NMR data sets were acquired on 10 urine samples from PDE patients and 19 samples from healthy controls, in an attempt to discriminate the two groups based on the presence of specific biomarkers, as previously reported. Under the conditions chosen for this study, the nhPHIP technique is selective for species carrying an α-amino acid and should, therefore, allow the detection of the PDE biomarkers identified in previous studies as derivatives of pipecolic acid (Figure 1). Figure 3 shows the H_I_ hydride region of a 1D nhPHIP and 2D nhPHIP ZQ NMR spectra acquired for one of the control urine samples. Note that the dynamic range spanned by these signals covers typically 2 orders of magnitude, which is much less than the range reported for conventional NMR spectra of urine. A large portion of the peaks in the 2D nhPHIP ZQ spectrum were previously assigned to proteogenic α-amino acids (Figure 3B), while several other signals originate from presently unassigned metabolites carrying an α-amino acidic group.

**Figure 3:**
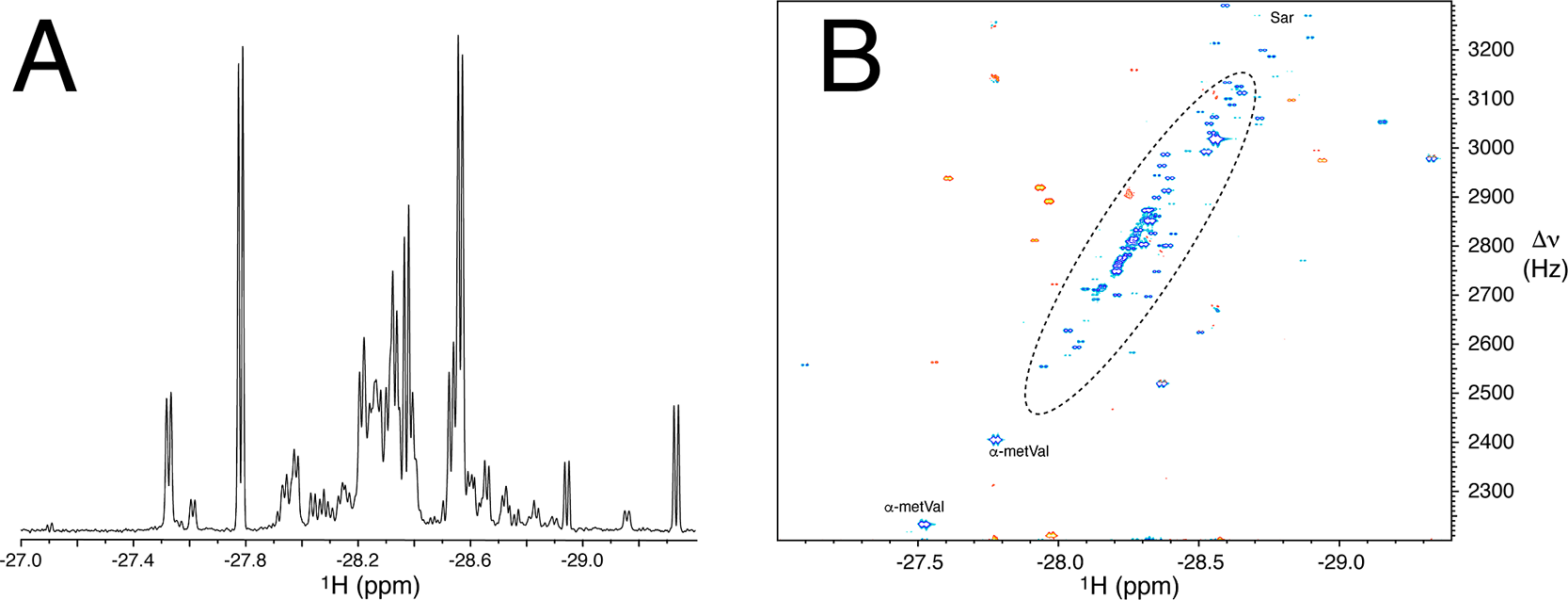
(A) H_I_ region of a 1D nhPHIP NMR spectrum of a sample of human urine (control) diluted 20 times in methanol. The spectrum was acquired in 2 m at 600 MHz ^1^H resonance frequency, in the presence of 0.85 mM Ir-IMes catalyst, 15 mM pyridine as cosubstrate, 20 mM triethylamine buffer, and 5 bar of 51% para-enriched H_2_ at 10 °C. (B) H_I_ region of a 2D nhPHIP ZQ NMR spectrum recorded in 1 h on the same sample. Signals enclosed in the elliptic dashed line originate from proteogenic α-amino acids. Signals in red are folded three times and have opposite phase compared to the signals in blue.

After standard NMR processing, uniform bucketing of 0.01 ppm width was applied to the 1D nhPHIP spectra. Principal Component Analysis (PCA) was initially used to explore the main trends of variation in the 1D nhPHIP data set, which resulted in complete separation between the two groups, with 26 and 16.5% variance explained by the first two principal components, as displayed in Figure 4A.

**Figure 4:**
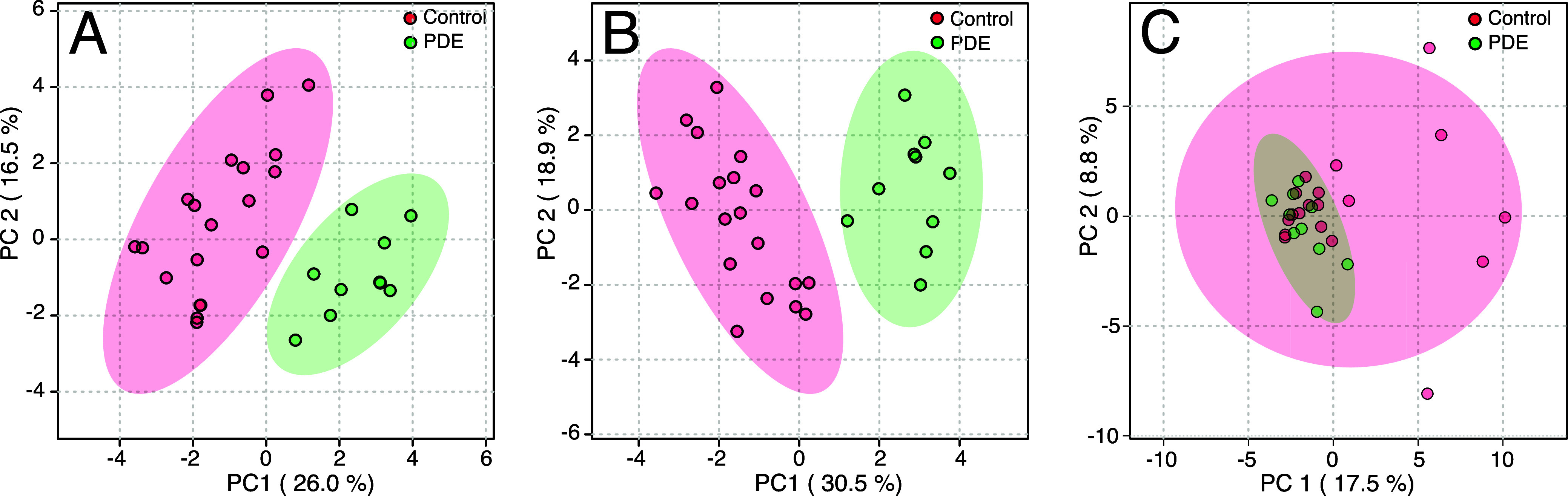
PCA 2D score plot for (A) the 1D nhPHIP data set, (B) the 2D nhPHIP ZQ data set, and (C) the conventional 1D ^1^H data set. All data sets were pretreated with log transformation and row-centering before PCA.

To explore the effect of enhanced resolution on the PCA result, the 2D nhPHIP data set recorded for the same urine samples was used. 2D NMR metabolomics offers the advantage of a higher resolving power, thereby reducing the signal overlap that can hamper group discrimination in multivariate analysis. When using conventional NMR, 2D spectroscopy comes at high costs in terms of sensitivity and reduced throughput, which makes its use less widespread in metabolomics. In the present case, however, no sensitivity penalty is associated with 2D nhPHIP ZQ spectroscopy. After standard NMR processing, the 2D spectra were manually integrated. In order to fully exploit the resolving power of the 2D spectra, only the integrals from well-resolved peaks were considered for the PCA, while signals in overlapping regions were discarded. Complete separation between the two sample groups was obtained, as shown in the 2D score plot in Figure 4B, with 30.5 and 18.9% variances explained by the first two principal components.

The statistical significance of the PCA separation for both nhPHIP data sets is indicated by a *p*-value < 0.001, as derived from a PERMANOVA test based on 999 permutations. Finally, untargeted NMR fingerprinting was performed using conventional 1D ^1^H NMR spectra for a comparison with a standard NMR-based metabolomics approach. As illustrated in Figure 4C, the 2D score-plot does not indicate any separation between the two sets of samples, independent of the applied data pretreatment.

In order to produce a predictive model for separating the PDE urine samples from controls, Partial Least Squares Discriminant Analysis (PLS-DA) was used. Other than PCA, PLS-DA is a supervised method in which sample classes are taken into consideration when building principal components. As displayed in Figure 5A–C, the score plots obtained from the PLS-DA of the three data sets indicate complete separation between the two classes along the first component.

**Figure 5:**
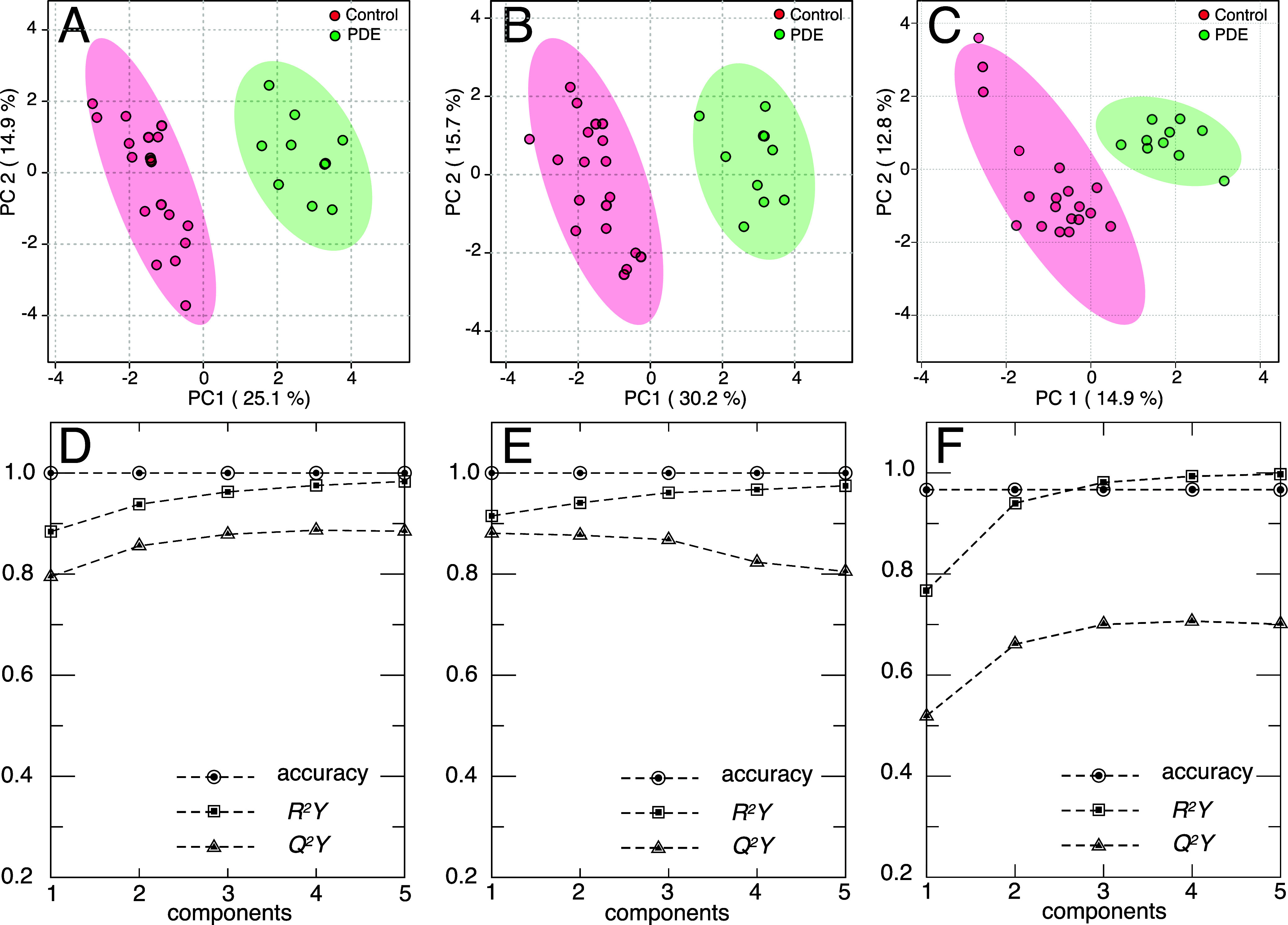
PLS-DA 2D score plot for (A) the 1D nhPHIP data set, (B) the 2D nhPHIP ZQ data set, and (C) the conventional 1D ^1^H data set. All data sets were pretreated with log transformation and row-centering before PLS-DA. Plots of the Performance Indicators (accuracy, *R* ^2^ *Y* and *Q* ^2^ *Y*) as a function of the number of employed variables for (D) the 1D nhPHIP data set, (E) the 2D nhPHIP data set, and (F) the conventional 1D ^1^H data set.

The percentages of variation between the samples explained by the first two components were 25.1 and 14.9% for 1D nhPHIP NMR, 30.2 and 15.7% for the 2D nhPHIP ZQ NMR, and 14.9 and 12.8% for conventional NMR data.

In Figure 5D–F, the performance curves for each one of the three models are displayed, reporting the values of accuracy, *R* ^2^ *Y* (goodness of fit) and *Q* ^2^ *Y* (predictive character) as a function of the number of the employed variables. The curves indicate a high accuracy and excellent predictive power for the model based on the nhPHIP data sets, while slightly lower values are observed for all Performance Indicators of the model built using the conventional NMR data.

In order to highlight the spectral features that best explain the differences between the two groups, variable importance in projection (VIP) was used for the nhPHIP and for the conventional ^1^H NMR data sets. The VIP scores indicate the importance of the related variables in the projections used for the PLS-DA model, where high values correlate with features that are important for group separation. The features with a VIP score higher than 1.5 are shown in Figure 6 for the two nhPHIP data sets.

**Figure 6:**
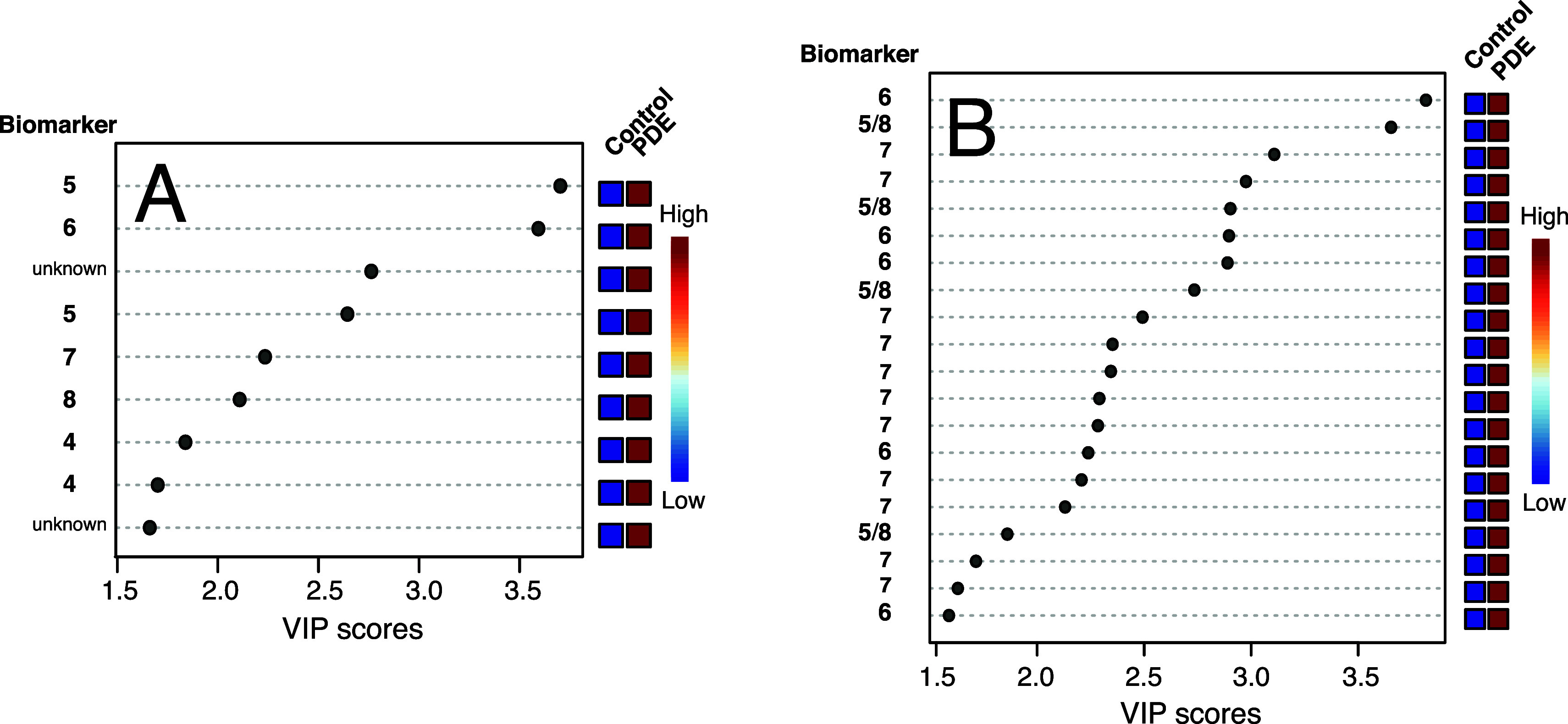
(A) VIP plot displaying the most important features in the PLS-DA separation with score >1.5 for the 2D nhPHIP data set. (B) VIP plot displaying the most important features in the PLS-DA separation with score >1.5 for the 1D nhPHIP data set. In each plot, the PDE biomarkers corresponding to the VIP features are indicated.

The plots in Figure 6 indicate a marked increase in the level of metabolites corresponding to the VIP features in the PDE samples compared to the control group. The identification of these putative biomarkers was attempted by spiking a urine sample from the control group with the biomarkers **4**, **6–10** (Figure 1). Thanks to the signal resolution and the spectral simplification of the 2D nhPHIP ZQ data set, this approach provided the unambiguous identification of five VIP features, as indicated in Figure 6A: two of them derive from pipecolic acid (**4**), one from 6-oxopipecolic acid (**6**), and the last two from (2*S*,6*S*) and (2*S*,6*R*)-6-(2-oxopropyl)­piperidine-2-carboxylic acid (**7, 8**).

To investigate the identity of the remaining four VIP features in plot 6A, the equilibrium between α-AASA (**2**) and Δ^1^-P6C (**5**) (Figure 1) was analyzed on a synthetic sample via a 2D nhPHIP ZQ experiment (see the Supporting Information). The most intense peaks of this artificial mixture were also observed in all PDE samples and were tentatively assigned to the diastereomeric complexes formed by Δ^1^-P6C (**5**). The last two VIP features in Figure 6A could not be assigned to any previously identified biomarker, or species formed in the synthetic equilibrium of α-AASA/Δ^1^-P6C. This observation is in agreement with recent works reporting about the presence of still unidentified PDE biomarkers in urine.^21^

VIP features could be identified with some of the PDE biomarkers signals also in the 1D nhPHIP data set, despite its lower resolution. Due to signal overlap, some features could be equally assigned to biomarker **5** or **8.** Note that a second signal for biomarker **7** ((2*S*,6*S*)-6-(2-oxopropyl)­piperidine-2-carboxylic acid) could be detected, which was not present in the 2D nhPHIP data set. Due to its line width, this signal is very weak in the high-resolution 2D nhPHIP spectra and was, therefore, discarded in the initial feature selection.

Finally, the VIP scores for the conventional ^1^H NMR data set were taken under consideration (Figure 7A). By overlaying the highest scoring features with the ^1^H NMR spectra of pure biomarkers (Figure 7B), it was possible to attribute some of them to biomarker **6**. This was not unexpected, as this biomarker has been reported as relatively abundant in urine (30–233 μm/mmol creatinine; see the Supporting Information for further details). A representative patient urine ^1^H spectrum in Figure 7B clearly displays several signals from biomarker **6**, which are not directly observable in the control urine spectrum. No other VIP feature could be associated with any of the PDE biomarker signals, possibly due to their substantially lower concentration in urine (see the Supporting Information) which, in combination with signal crowding, makes their detection challenging.

**Figure 7:**
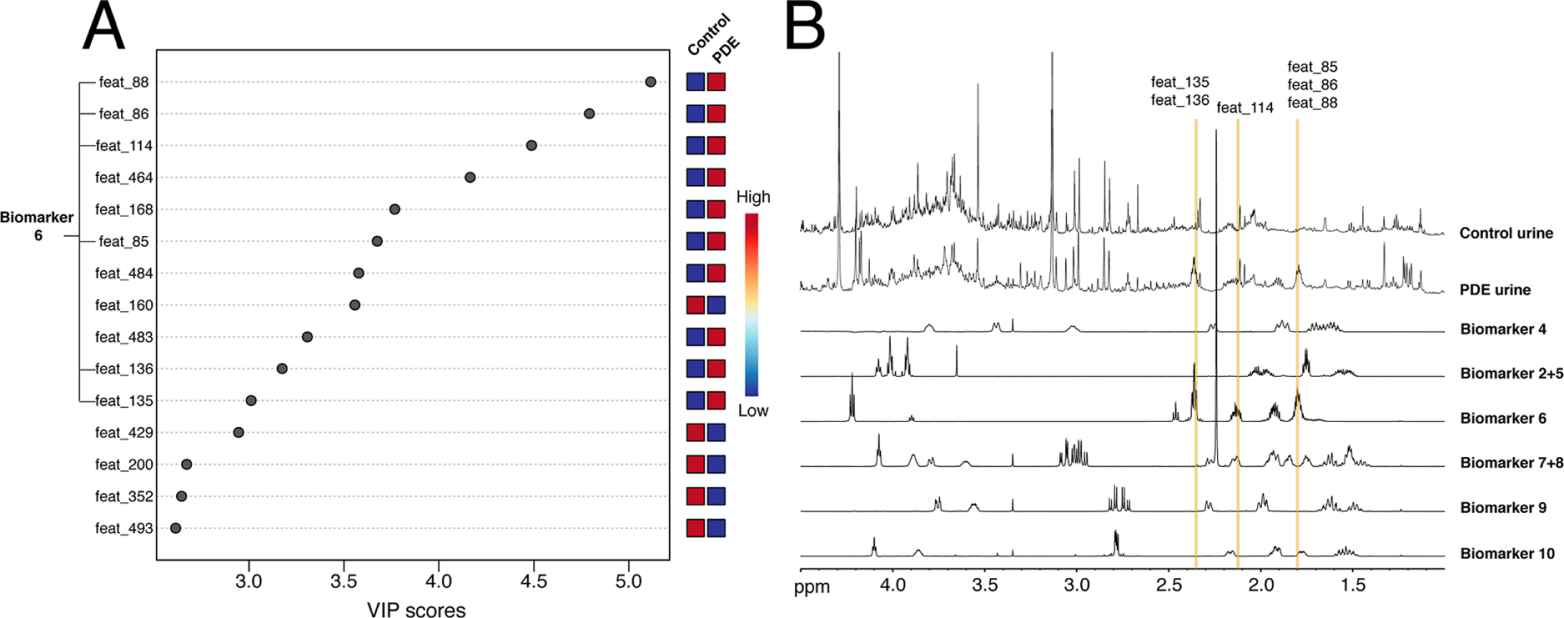
(A) VIP plot displaying the most important features in the PLS-DA separation with score >2.5 for the ^1^H NMR conventional data set. (B) Overlay of the aliphatic region of the ^1^H NMR spectra of the PDE biomarkers, together with two ^1^H urine spectra, from the patient and the control group, respectively. All spectra were recorded in the same conditions in H_2_O/D_2_O 90:10 (v/v) at pH 2.5, 298 K at 600 MHz ^1^H resonance frequency. The correspondence between some of the VIP features displayed in (A) and the biomarkers signals is indicated.

Finally, to estimate nhPHIP sensitivity in this study, biomarker **7** was quantitatively determined in one of the PDE urine samples by single point addition. From the concentration in the nhPHIP NMR sample (2.0 ± 0.1 μM), a limit of detection of 70 nM can be estimated for biomarker **7**, corresponding to 1.4 μM in urine (see the Supporting Information). Our determination (3.4 ± 0.2 μM/mM creatinine) is consistent with a previous LC–MS/MS determination (3 ± 2 μM/mM creatinine) of the total concentration of the two diastereoisomeric biomarkers **7** and **8** in a set of eight urine samples from PDE patients.^21^

## Discussion

Over the last years, nhPHIP NMR chemosensing has proven to be a valuable tool for the investigation of dilute compounds in complex mixtures. While the technique itself carries little structural information, the robust protocols developed in the last decades allow the use of chemical shift databases for a reliable metabolite identification based on the hydride resonances. In addition, previous applications have demonstrated that quantitative information can be extracted from nhPHIP data, e.g., allowing pharmacokinetic studies on nicotine metabolites in urine,^28^ as well as the determination of enantiomeric excess in complex mixtures.^13^ Because of its enhanced sensitivity and resolution, nhPHIP can prove an important tool for metabolomics, complementary to conventional ^1^H NMR spectra. As already pointed out, the selective character of nhPHIP makes it suitable for semi-targeted studies, in which only specific classes of compounds are detected. In the application presented here, this resulted in the selective observation of metabolites carrying an α-amino acid group, producing a considerable spectral simplification and more homogeneous concentration range, compared to conventional ^1^H NMR spectroscopy of urine. These features, together with lower NMR detection limits, resulted in a superior sensitivity to metabolic composition, as evidenced by the results of a multivariate analysis on the nhPHIP data sets. Although the acquisition of 2D spectra can greatly enhance signal resolution, it is not often used in NMR-based metabolomics because of the lower sensitivity and generally longer experimental times.^4^ However, nhPHIP hyperpolarization allows the measurement of 2D NMR spectra with submicromolar sensitivity and a high signal resolution. VIP analysis of the nhPHIP data clearly indicates that group separation is due to a limited number of signals that selectively appear in the urine samples of PDE patients. nhPHIP measurements on a spiked urine sample allowed to associate most VIP features to previously determined PDE biomarkers. As for the conventional ^1^H data set, only biomarker **6**, previously reported as relatively abundant in urine from PDE patients, was identified based on the analysis of the VIP features, while all the others appear to suffer from excessive dilution in combination with signal crowding. This clearly demonstrates how nhPHIP can complement conventional NMR metabolomics by highlighting biomarkers that are too dilute and/or suffer from signal overlap in conventional ^1^H spectra. The superior biomarkers coverage attained via nhPHIP is particularly important in the present case, as only a subset of the listed metabolites (i.e., **7**, **8**, **9**, and **10**) have been reported as truly unique to PDE, while biomarker **6** (the one highlighted by conventional ^1^H data) has been also associated with other metabolic disorders.^29^

## Conclusion

We have herein presented the first implementation of para-hydrogen-based hyperpolarization in an NMR metabolomics study. Our results clearly indicate that nhPHIP NMR is sufficiently mature to be employed in metabolomics. The benefits provided by this technique include a straightforward sample preparation, the access to a more dilute portion of the metabolome and a solution to signal crowding that hampers biomarker detection in conventional ^1^H NMR studies. Due to the selective nature of the technique, nhPHIP should be considered as a complementary tool to standard ^1^H NMR spectra, routinely employed in NMR-based metabolomics. Its inherent semi-targeting character warrants, therefore, a careful evaluation of the research question whereupon it is applied. Note, however, that although the protocol employed for this study is tailored for α-amino acids, different conditions can be applied to focus on other metabolite species, such as nucleotides, amines, and heteroaromatic compounds.^25^

In order for nhPHIP to become a standard tool in NMR-based metabolomics, we envision that a few issues need to be properly addressed. In the first place, comprehensive databases of hydride chemical shifts must be built for metabolite identification. This requirement is not unique for nhPHIP, as similar databases are routinely employed in conventional ^1^H NMR metabolomics. In addition, although the nhPHIP 2D ZQ spectra employed here suffer no sensitivity penalty, they require relatively long acquisition times (ca. 1 h per sample) to achieve the desired resolving power, which is not compatible with the sample throughput demanded in metabolomics. Recent developments, however, have demonstrated that 2D nhPHIP NMR can be measured more efficiently, resulting in a much faster acquisition.^30^ When combined with modern compressed sensing techniques, as previously demonstrated,^31^ nhPHIP NMR can be proven fully compatible with the high throughput requirements of a metabolomics study.

## Supplementary Material



## Notes

Material and methods, PDE biomarker structure and reported concentrations in urine, ^1^H NMR spectra of urine and of PDE biomarkers, chemical shifts of hydride signals of PDE Biomarkers complexes, feature selection of 2D ZQ spectra, Limit of Detection for biomarker **7**, and an ethical statement^32–37^ (PDF)
