# Supplementary material for: Semi-Targeted Nuclear Magnetic Resonance Metabolomics via Parahydrogen-Induced Hyperpolarization for Enhanced Sensitivity to Metabolic Composition
Source: J Am Chem Soc. 2025 Aug 26;147(36):33185–92. doi: 10.1021/jacs.5c11226 (PMC12426882; doi:10.1021/jacs.5c11226)
Supplement: Supplementary file 1 [file ja5c11226_si_001.pdf]

Supporting Information

Semi-Targeted Nuclear Magnetic Resonance Metabolomics via Parahydrogen-Induced Hyperpolarization for enhanced sensitivity to metabolic composition

Thom B. Posthumus[a], Udo F. H. Engelke[b], Ruud L.E.G. Aspers[a], Jona Merx[a], Thomas J. Boltje[a], Jonathan Martens[a], Ron A. Wevers[b], Martin C. Feiters[a], Floris P. J. T. Rutjes[a], Marco Tessari[a,]*

[a] Institute for Molecules and Materials

Radboud University

Heyendaalseweg 135, 6525 AJ Nijmegen, the Netherlands

E-mail: Marco.Tessari@ru.nl

[b] Translational Metabolic Laboratory, Department Human Genetics

Radboud University Medical Centre

Geert Grooteplein Zuid 10, 6525 GA Nijmegen, the Netherlands

Contents

Chemicals and Materials 3
nhPHIP 4
NMR acquisition and processing 5
Quality Control 6
Data treatment 6
PDE biomarkers: structure, name and reported concentrations in patient urine 7
^{1}H NMR Spectra of urine and PDE biomarkers 8
Chemical shifts of hydride signals of PDE biomarkers complexes 9
2D nhPHIP ZQ spectrum of a PDE urine sample. 11
Limit of detection: biomarker 7 12
Ethical statement 14
SI References 14

Chemicals and Materials

Chemicals

All chemicals were used as supplied. The catalyst precursor [Ir(COD)(IMes)Cl](Imes = 1,3-bis(2,4,6-trimethylphenyl)imidazole-2-ylidene; COD = cyclooctadiene) was synthesized in-house according to published methods.[1]

Biomarkers **7**-**10** were synthesized according to the published protocol.[2]

Pyridine, triethylamine hydrochloride, piperidine, piperidine hydrochloride, (*R*)-(+)-α-methylvaline, hydrochloric acid 37%, deuterated water with 0.05% TSP, sodium hydroxide were purchased from Sigma-Aldrich. L-Proline was purchased from Sigma Chemical Company. Methanol was purchased from Fischer Scientific. Biomarker **6** was purchased from Fluorochem. L-Allysine ethylene acetal was obtained from DSM. Triethylamine was purchased from Acros Organics.

Ultrapure water (MilliQ) was generated by a Milli-Q academic water purification system with a Q-Gard® 2 purification pack and a Quantum® EX cartridge purchased by Millipore.

A parahydrogen generator (HyperSpin Scientific) cooled by liquid nitrogen at 77 K was used to convert thermal hydrogen (purity 5.0, Linde Gas Benelux B.V.) to 51% *para*-enriched H_2.

Urine collection and storage

Nineteen age-matched control samples were obtained from leftover material from subjects not suffering from metabolic disorders and not showing abnormalities in targeted-metabolite analysis. 10 PDE-ALDH7A1 patient samples, belonging to 10 different patients, were previously collected for routine metabolic screening or treatment follow-up. All samples were stored at -20 °C prior to analysis.

Sample preparation for thermal ^1H NMR

The urine samples were thawed and vortexed. A volume of 800 μL or 1 mL was transferred to a vial and the pH was set to 2.5 ± 0.1 using concentrated HCl. After centrifugation at 13000 rpm for 5 minutes, 700 μL of the supernatant was transferred to a vial containing 100 μL D_2O with 0.05% TSP (v/v). The sample was vortexed and transferred to a NMR sample tube.

Sample preparation for nhPHIP

Urine

The urine samples were thawed and vortexed. 40 μL of urine was transferred to a vial containing 850 μM Ir-IMes catalyst precursor, 15 mM pyridine, 20 mM triethylamine buffer at pH 10.8 and 10 μM methylvaline as internal reference for a total volume of 800 μL MeOH/H_2O with 6.25% water content (v/v). The mixture was then vortexed and centrifuged for 5 minutes at 13000 rpm. Subsequently, a sample aliquot of 650 μL was transferred to a Wilmad® QPV NMR tube with 5 mm diameter.

All in all, the original urine samples were diluted 20 times in methanol for the nhPHIP NMR measurements.

Note that no ^2H lock was employed in the acquisition of nhPHIP spectra.

Biomarkers nhPHIP samples
For each individual biomarker (except **2** and **5**, see below) a solution was prepared containing 100 µM Ir-IMes catalyst precursor, 1 mM pyridine, 10 mM piperidine buffer at pH 11.1, biomarker (50 µM) and L-proline (50 µM) as internal standard, for a total volume of 800 µL in MeOH with 5% H_2O content (v/v). The mixture was then vortexed and centrifuged for 5 minutes at 13000 rpm. Subsequently, a sample aliquot of 650 µL was transferred to a Wilmad® QPV NMR tube with 5mm diameter.

*Incubation of α-AASA /Δ^1-P6C (**2, 5**)*
In order to prepare the reactive mixture of biomarkers **2** and **5**, HCl (36 µL, 0.01M) was added to L-allysine ethylene acetal (36 µL, 10 mM) in H_2O, and the mixture was vortexed for 60 minutes. Afterwards, the pH was neutralised using dilute aqueous NaOH. The final total concentration of **2** and **5** was 3.3 mM.

*Biomarkers **2** + **5** nhPHIP samples*
A sample was prepared containing 300 µM Ir-IMes catalyst precursor, 4.5 mM pyridine, 6.0 mM piperidine buffer at pH 11.1, 150 µM of biomarkers **2** and **5** and 10 µM L-proline as internal reference for a total volume of 800 µL MeOH with 6.25% H_2O content (v/v). The mixture was then vortexed and centrifuged for 5 minutes at 13000 rpm. Subsequently, a sample aliquot of 650 µL was transferred to a Wilmad® QPV NMR tube with 5mm diameter.

nhPHIP

Samples were transferred into a quick pressure valve NMR tube (Wilmad® 528-QPV-7) and connected to an in-house designed hydrogen 'bubble set-up'. After flushing the sample for a few minutes with nitrogen to reduce dissolved oxygen, *p*-H_2 was bubbled through the solution to activate the catalyst. The tube was then placed in a warm water bath at 50 °C for 7.5 minutes while maintaining a pressure of 5 bar *p*-H_2 to accelerate the attainment of the PHIP-active binding mode for α-amino acids. The tube was then cooled down to 5 °C for 2 minutes before placing the sample in the magnet of the NMR spectrometer at 10 °C.
At the beginning of each transient, the NMR sample was saturated with *p*-H_2. This process involves four steps under spectrometer control:
(1) the pressure in the NMR tube is reduced from 5 to 4 bar via a relief valve connected to the vent line (0.25 s),
(2) *para*-enriched H_2 is bubbled through the solution, restoring the tube pressure to 5 bar (0.75-1.5 s),
(3) after closing the bubbling valve, pressure is applied above the liquid (0.3 s). This helps stabilizing the pressure, preventing any residual H_2 bubble passing through the solution during the 2D pulse sequence,
(4) NMR solution is rested (0.7-1.0 s) before starting the actual pulse sequence, to dampen the turbulent motions resulting from bubbling.

NMR acquisition and processing

All NMR spectra presented in this work were acquired on a Bruker Avance III spectrometer operating at 600 MHz ^{1}H resonance frequency, with a HCN triple-resonance cryo-cooled probe equipped with z-pulsed field gradients.

Thermal 1D ^{1}H NMR

Thermal ^{1}H 1D spectra were acquired at 25 °C using a 1D NOESY experiment (noesygppr1d) with water presaturation. An acquisition time of 2.185 s for a spectral width of 15000 Hz was employed. Sixteen dummy scans were run before acquisition started, and the signal was accumulated with 128 transients with a recycling delay of 4 s for a total time of 15 min per sample. An exponential apodization function (line broadening 0.5 Hz) was applied prior to zero filling to 128k points and Fourier transform. Phase and baseline were manually corrected using nmrPipe.[3] Chemical shifts were referenced with respect to TSP.

Thermal 1D ^{1}H NMR of hydride region

Hydride spectra under thermal equilibrium conditions were acquired with 128 transients using excitation sculpting to suppress the solvent signals, with 6 s recovery delay, a spectral width of 8400 Hz and 0.49 s acquisition time. A Lorentz-to-Gauss apodization function was applied prior to zero filling to 32k points and Fourier transform. Phase and baseline were manually corrected using nmrPipe.

nhPHIP NMR

All hydride nhPHIP spectra were acquired at 10 °C.

Hydride nhPHIP 1D NMR signals with 8400 Hz spectral width were acquired for 0.49 s after 8 dummy scans, with 32 transients using a SEPP (selective excitation of polarization using PASADENA) pulse scheme, centered approximately at -26 ppm for a total time of 2 minutes. A Lorentz-to-Gauss apodization function was applied prior to zero filling to 32k points and Fourier transform. Phase and baseline were manually corrected using nmrPipe.

Each 2D ZQ nhPHIP hydride spectrum was acquired in 1 h, with 2 transients per increment, 768 real increments, 8400 Hz and 2200 Hz spectral width in the direct and indirect dimension, respectively. Note that, in order to minimize the overall acquisition time, folding of the 2D signals in the indirect dimension was employed. The 2D data sets were processed with nmrPipe using Lorentz-to-Gauss apodization in both dimensions, prior to zero filling to 2048(t_1) × 32768(t_2) and Fourier transformation. Correction due to the field drift was implemented by signal alignment before Fourier Transform in the indirect dimension. Removal of t_1-noise in 2D ZQ spectra in correspondence of the most intense hydride signals was achieved by applying the method of Wei et *al*.[4]

Quality Control

nhPHIP samples containing all constituents except urine were prepared as described and measured to determine the extent of background signals and exclude cross contamination between successive experiments. For extra safety, randomized acquisition of samples from control and patient groups was avoided.
No quality control samples were used to assess the long-term repeatability of the nhPHIP measurements.

Data treatment

Thermal 1D ^{1}H NMR dataset
Spectral alignment with a least squares algorithm and bucketing were performed on NMRPROCFLOW[5], using intelligent bucketing of the spectral ranges 0.6-4.7 ppm, 5.1-5.5 ppm, 6.1-10.0 ppm with SNR ratio 5, yielding 529 buckets.

1D nhPHIP NMR dataset
Uniform bucketing was employed in the "H_I" hydride region for α-amino acids (between –27.3 and -29.3 ppm) that is typically better resolved, resulting in 223 bins with 0.008 ppm width and integral larger than 5 times the noise level for at least 33% (i.e. 10) of the samples.

2D ZQ nhPHIP NMR dataset
Well-resolved 2D peaks from the "H_I" hydride region between –27.3 and -29.3 ppm were integrated using hand marked regions. Selection of the signals with integrals larger than 5 times the noise level yielded 73 features (see 2D spectrum at page 9).

Prior to statistical analysis using MetaboAnalyst 6.0[6] the three datasets were subjected to the same treatment (i.e. log transformation and sample centering).
Alternative approaches commonly employed in NMR metabolomics (e.g. total sum normalization combined to centering and Pareto- or autoscaling) were attempted on the three datasets but did not result in any separation between the two sample groups.

PDE biomarkers: structure, name and reported concentrations in patient urine

Table S.1. Structure, name and concentration range of PDE biomarkers. *The range for Biomarker 7 and 8 refers to their summed concentrations.

Biomarker	Concentration average and range (µmol/mmol Creatinine)
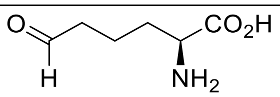 α-aminoadipic semialdehyde (α-AASA, **2**)	31.4 (1.8 – 94.3)[7]
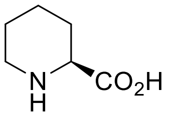 pipecolic acid (**4**)	0.24 (0.02 – 0.38)[7]
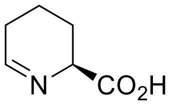 Δ1-piperideine-6-carboxylic acid (**5**)	20.5 (1.41 – 27.66)[7]
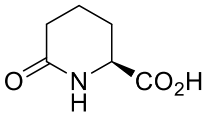 6-oxopipecolic acid (**6**)	117.4(30-233)[8]
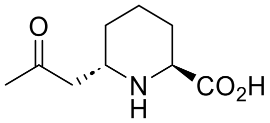 (2S,6S)-6-(2-oxopropyl)piperidine-2-carboxylic acid (**7**)	2.6 (0.3-4.4) [8,*]
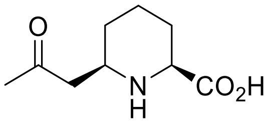 (2S,6R)-6-(2-oxopropyl)piperidine-2-carboxylic acid (**8**)	2.6 (0.3-4.4)[8,*]
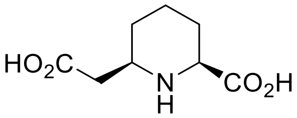 (2S,6R)-6-(carboxymethyl)piperidine-2-carboxylic acid) (**9**)	No data available
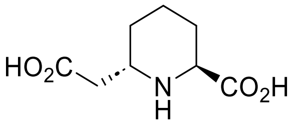 (2S,6S)-6-(carboxymethyl)piperidine-2-carboxylic acid) (**10**)	No data available

1H NMR Spectra of urine and PDE biomarkers

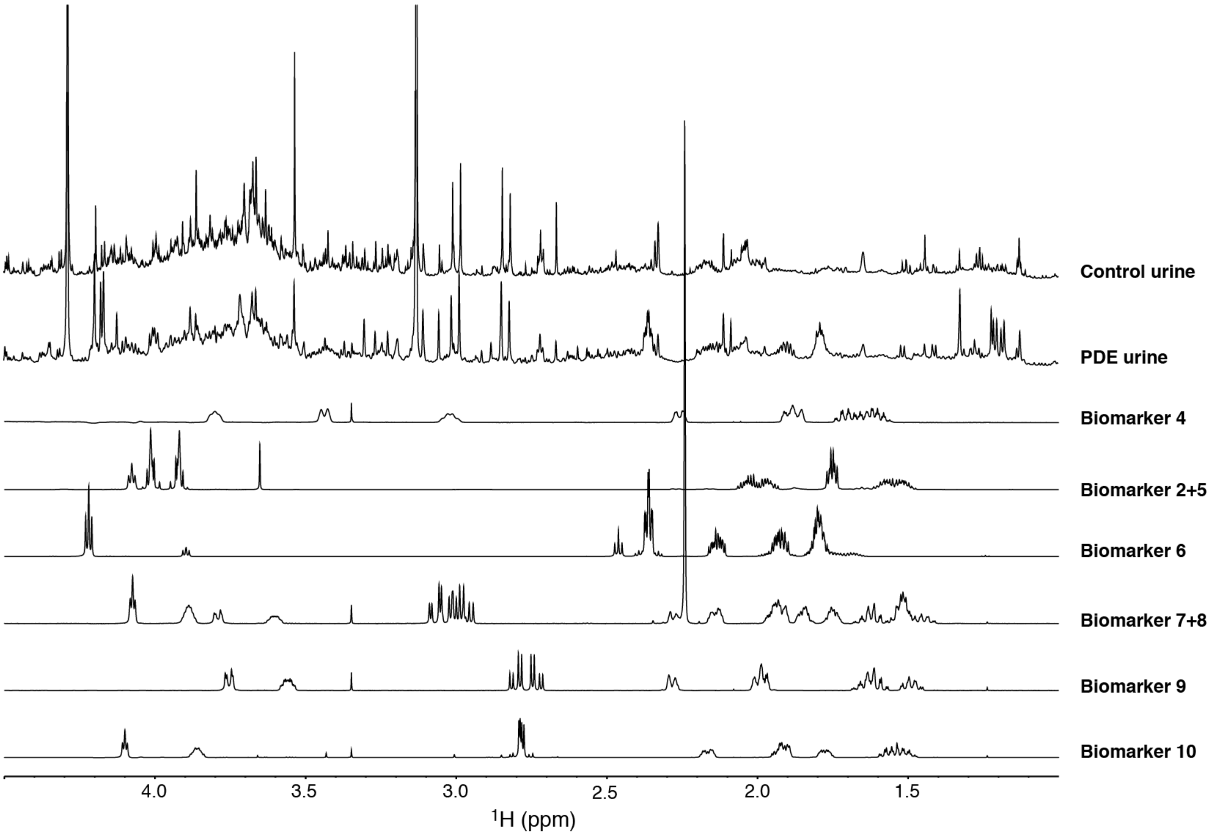

Figure S1: Overlay of the aliphatic region of the ^1H NMR spectra of the PDE biomarkers, together with two ^1H urine spectra, from the patient and the control group, respectively. All spectra were recorded in the same conditions in H_2O/D_2O (90%/10%) at pH 2.5, 298 K at 600 MHz ^1H resonance frequency

Chemical shifts of hydride signals of PDE biomarkers complexes

Table S.2. Chemical shifts of the hydride signals of the PDE biomarkers complexes measured at 10 °C in $CH_3OH:H_2O$ 94:6 (v/v).

Biomarker	H_S hydride (ppm)	H_I hydride (ppm)
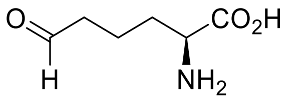 **2**	-23.59	-28.32
	-23.65	-28.18
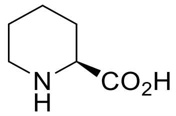 **4**	-23.37	-29.47
	-23.42	-28.57
	-23.85	-28.53
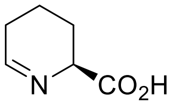 **5**	-22.92	-27.79
	-23.05	-27.75
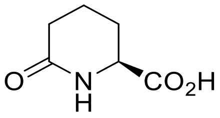 **6**	-21.54	-27.44
	-23.59	-28.24
	-23.60	-28.10
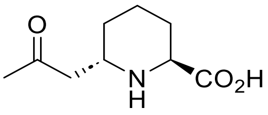 **7**	-23.54	-29.05
	-23.63	-29.23
	-23.74	-27.98
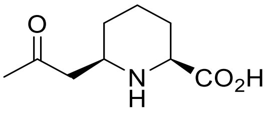 **8**	-23.84	-27.75
	-24.08	-28.83
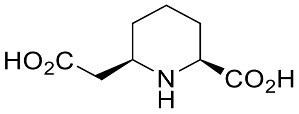 **9**	-23.77	-27.50
	-23.77	-27.77
	-23.94	-28.82
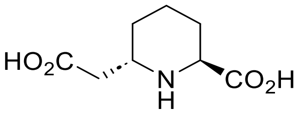 **10**	-23.2	-29.18
	-23.68	-29.03
	-23.83	-28.75

The assignment of the hydrides for the complexes formed by biomarkers **2** and **5** is hampered by the dynamic equilibrium between the two species, sketched below:

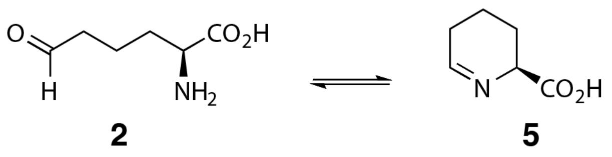

The tentative assignment presented in Table S.1 is based on the similarity between the structures of biomarker **2** and α-aminoadipic acid. Figure S2 below displays an overlay of the H$_I$ hydride regions of the 2D nhPHIP ZQ spectra recorded on the mixture of biomarkers **2** and **5** and on α-aminoadipic acid.

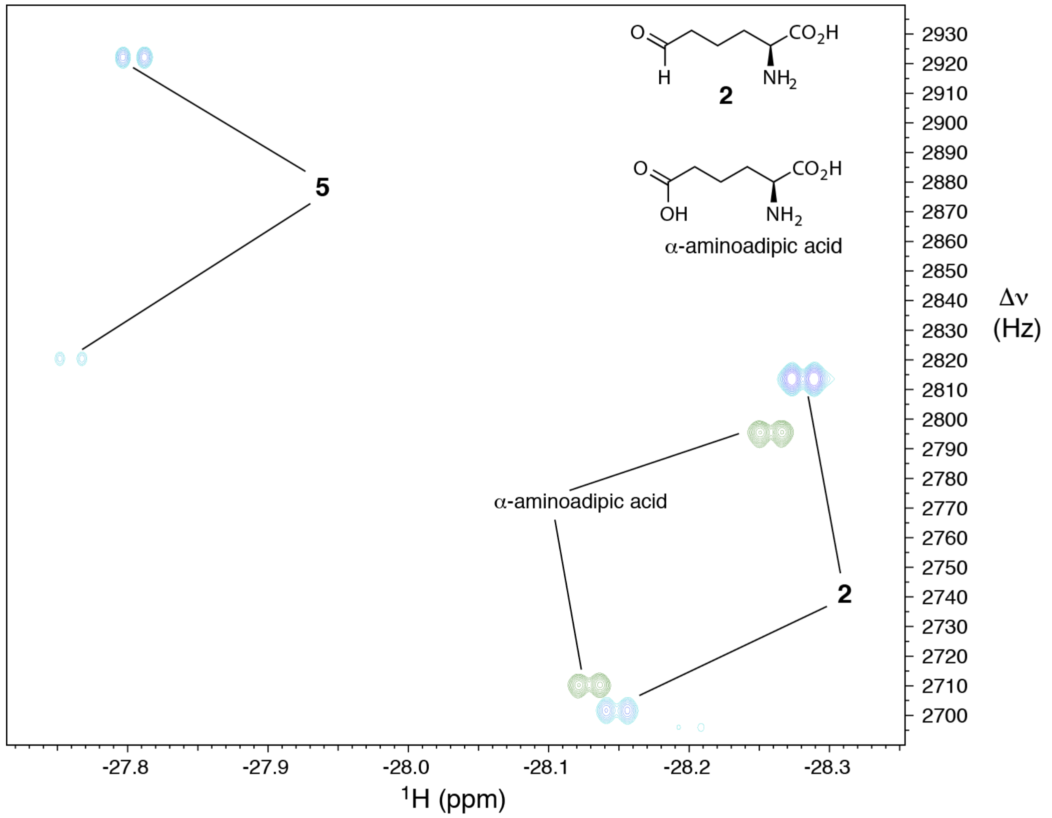

Figure S2: Overlay of the H$_I$ hydride region of the 2D nhPHIP ZQ spectra of the biomarkers mixture of **2** and **5** (blue peaks) and of α-aminoadipic acid (green peaks). The slight differences in chemical shift compared to the values reported in Table S.1 are due to somewhat higher water content in this biomarker mixture.

2D nhPHIP ZQ spectrum of a PDE urine sample.

The 2D nhPHIP ZQ spectrum of a urine sample from a PDE patient in the figure below provides an overview of the peaks included in the multivariate analysis.

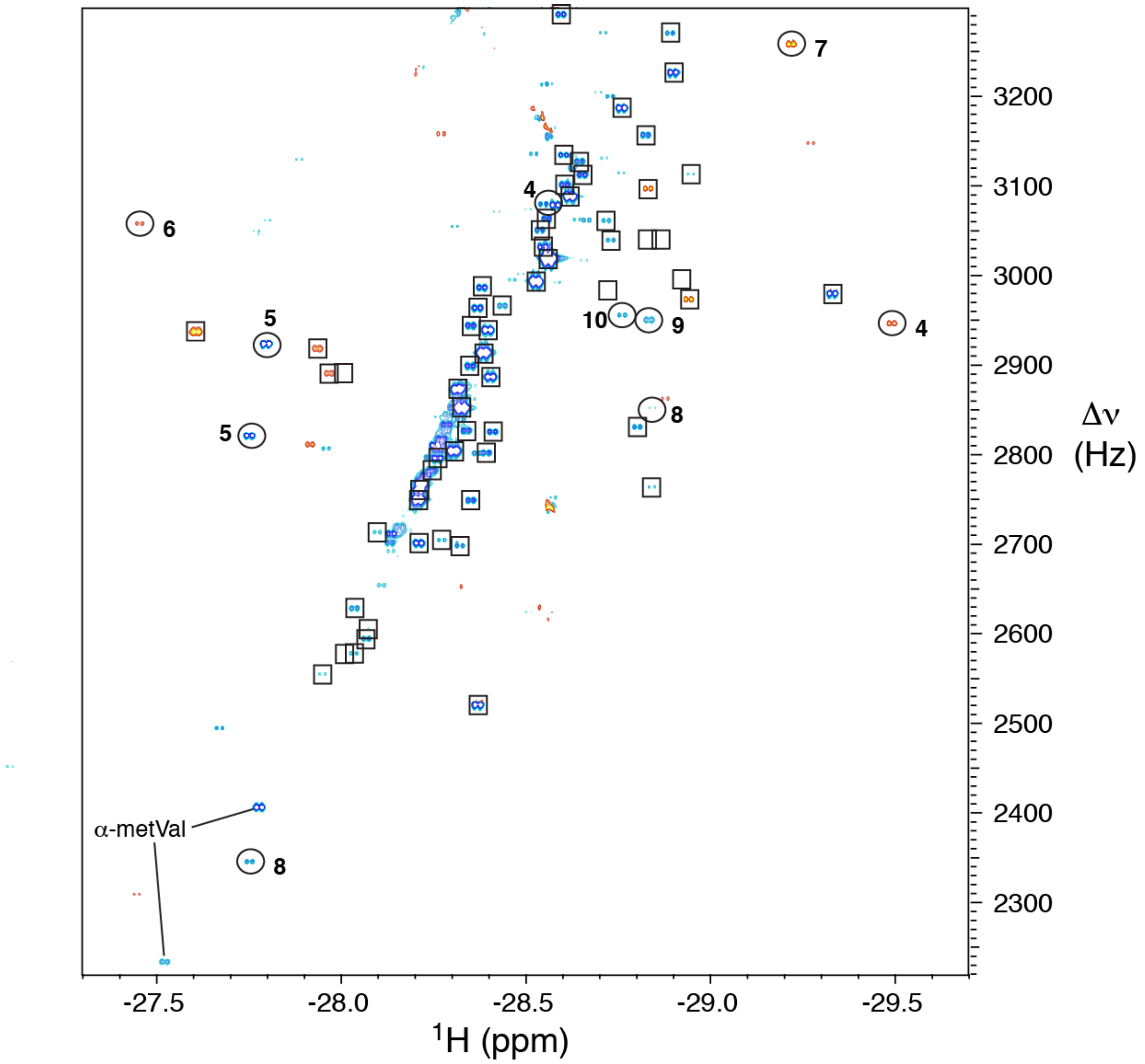

Figure S3: H_I hydride region of the 2D nhPHIP ZQ spectrum acquired on a PDE urine sample at 10 °C in MeOH:H_2O 94:6 (v/v). Square or circular boxes indicate the 2D signals considered for multivariate analysis. Peaks of resolved PDE biomarkers are enclosed with circles and their assignment is indicated. α-methylvaline was added as an internal reference and, therefore, its signals were not considered for multivariate analysis.

Limit of detection: biomarker 7

In order to establish the sensitivity of nhPHIP, biomarker **7** was quantitatively determined by single point addition in one of the PDE urine samples. Analogous to standard addition, this approach requires a linear dependence between analyte concentration and (hyperpolarized) signal integral. As previously demonstrated[9], such linearity holds in the presence of an excess of iridium catalyst with respect to amino acids in solution. This can be experimentally verified by recording a hydride spectrum under thermal equilibrium conditions. The presence of the hydride signal corresponding to the symmetric complex resulting from the association of the two units of co-substrate (i.e. pyridine) in the equatorial plane of the complex indicates an excess of catalyst with respect to α-amino acid ligands, as shown in Figure S4 below.

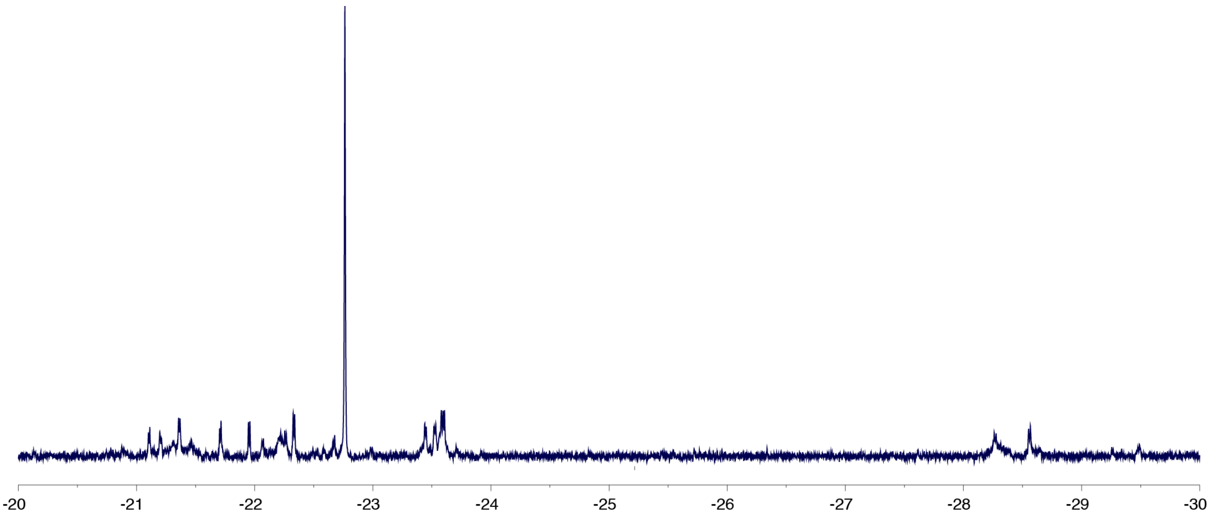

Figure S4: Hydride spectrum of the PDE sample used for the concentration determination of biomarker **7.** The large singlet at -22.74 ppm originating from the symmetric complex with two pyridine ligands in the equatorial plane amounts to ca. 25% of the total iridium catalyst in solution.

Figure S5 below displays the overlay of the nhPHIP ZQ and 1D nhPHIP spectra of the NMR sample from PDE urine and of a second NMR sample prepared in the same way, except for the addition of 4.89 μM of biomarker **7** (gravimetrically determined).
In order to account for potentially different levels of PHIP enhancement in the two samples, the signal integrals of a number of resolved 2D peaks, with intensity well above the noise level, were determined (excluding those of the spiked biomarker **7**). An average value α = 1.03 +/- 0.06 was calculated, indicating comparable PHIP enhancements in the two spectra.
The following expression holds between the integral ratio in the two spectra and the concentration of **7** in the NMR sample:

$$\alpha \frac{C_0 + \Delta C}{C_0} = \frac{I}{I_0} = R_7 \qquad \text{(s.1)}$$

where C_0 and ΔC indicate the concentration of **7** in the original sample and the spiked concentration (4.89 μM), respectively. I_0 and I indicate the integral of the nhPHIP signal of **7** in the original NMR urine sample and in the spiked sample, respectively.

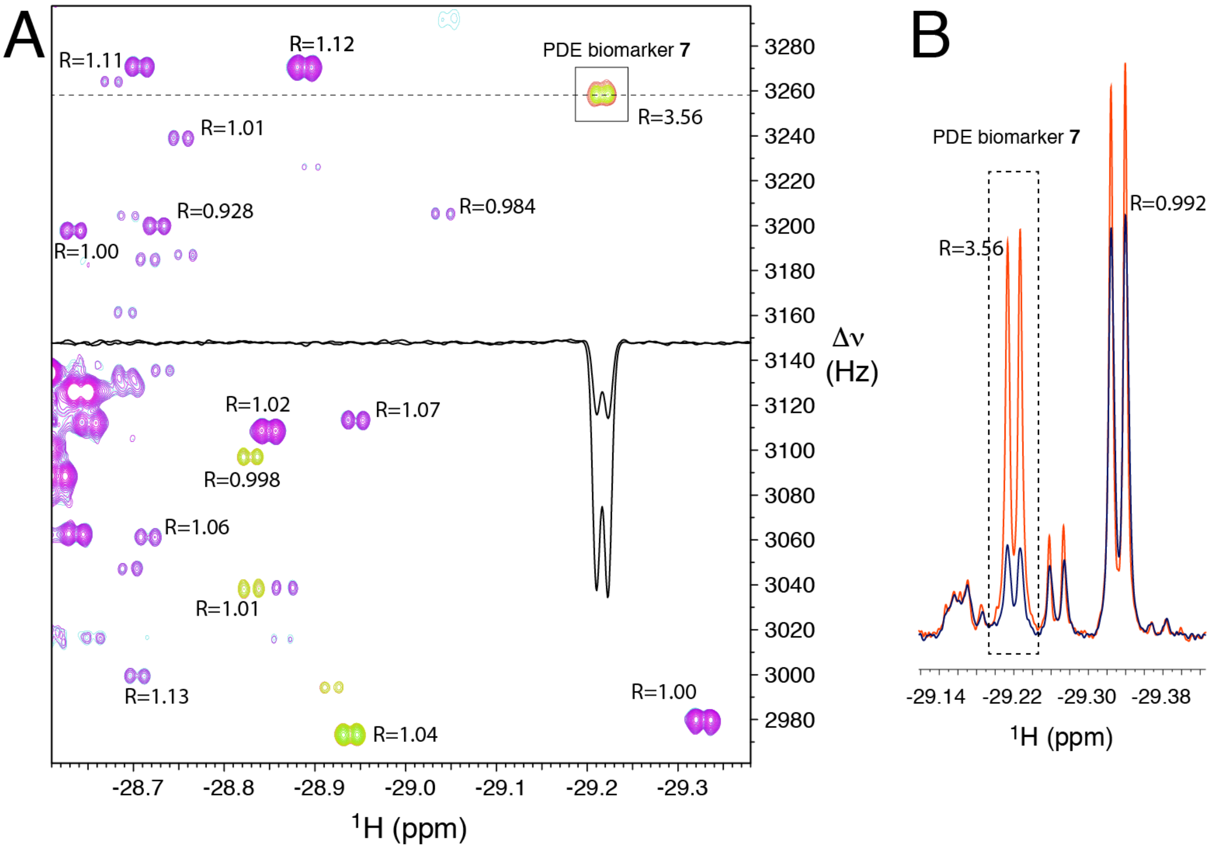

Figure S5: **(A)** Overlay of a portion of the 2D nhPHIP ZQ spectrum for a PDE urine sample, without (violet and green peaks) and with addition of 4.89 μM biomarker **7** (blue and red). The value of the integral ratio for resolved signals with intensity well above the noise level is reported. The signal used for the quantification of biomarker **7** is boxed. The 1D traces corresponding to the signal of **7** used for quantification are displayed. **(B)** Overlay of a portion of the 1D nhPHIP spectrum for the same urine samples as in (A): PDE urine sample (blue) and after addition of 4.89 μM biomarker **7** (red). The signal used for the quantification of **7** is boxed. Note that the intensity variation observed for the other signals derives from a different lineshape (e.g. results from different shimming quality) in the two spectra, while the integral ratio approaches 1 within experimental precision.

By rearranging eq. S.1 the concentration of **7** in the NMR urine sample can be estimated as:

$$C_0 = \frac{\alpha \Delta C}{R_7 - \alpha} \pm \frac{R_7 \Delta C}{(R_7 - \alpha)^2} \Delta\alpha = \frac{1.03 \times 4.89 \times 10^{-6}}{3.56 - 1.03} \pm \frac{3.56 \times 4.89 \times 10^{-6}}{(3.56 - 1.03)^2} \times 0.06 = 2.0 \pm 0.1\ \mu M$$

Considering the creatinine level (11.9 mM) and the 20-fold dilution in the NMR sample, this value corresponds to a concentration of 3.4 $\pm$0.2 μM / mM creatinine in urine, to be compared with 3 $\pm$2 μM / mM creatinine for the summed concentration of diastereoisomers **7**+**8**, as derived from LC-MS/MS.[8]
Based on the signal-to-noise ratio for the signal of **7** in the 2D ZQ spiked spectrum (300:1), a limit of detection of 70 nM in the NMR sample can be estimated, corresponding to 1.4 μM in urine.

Ethical statement

All patients and control subjects (or their guardians) registered their informed consent for the possible use of their leftover body fluid samples from clinical diagnostics for laboratory method validation purposes in their electronic patient record, in agreement with institutional and national legislation, as reviewed by the accredited Research Ethics Committee of Radboud University Medical Centre (file number 2021–7296).

SI References

(1) Kelly Iii, R. A.; Clavier, H.; Giudice, S.; Scott, N. M.; Stevens, E. D.; Bordner, J.; Samardjiev, I.; Hoff, C. D.; Cavallo, L.; Nolan, S. P. Determination of N-Heterocyclic Carbene (NHC) Steric and Electronic Parameters using the [(NHC)Ir(CO)2Cl] System. *Organometallics* **2008**, *27* (2), 202-210. DOI: https://www.doi.org/10.1021/om701001g
(2) van Outersterp, R. E.; Engelke, U. F. H.; Merx, J.; Berden, G.; Paul, M.; Thomulka, T.; Berkessel, A.; Huigen, M. C. D. G.; Kluijtmans, L. A. J.; Mecinović, J.; et al. Metabolite Identification Using Infrared Ion Spectroscopy—Novel Biomarkers for Pyridoxine-Dependent Epilepsy. *Anal. Chem.* **2021**, *93* (46), 15340-15348. DOI: https://www.doi.org/10.1021/acs.analchem.1c02896
(3) Delaglio, F.; Grzesiek, S.; Vuister, G. W.; Zhu, G.; Pfeifer, J.; Bax, A. NMRPipe: a multidimensional spectral processing system based on UNIX pipes. *J. Biomol. NMR* **1995**, *6* (3), 277-293. DOI: https://www.doi.org/10.1007/bf00197809
(4) Wei, S.; Ding, Y.; Song, K.; Liu, Z. A robust t1 noise suppression method in NMR spectroscopy. *Magn. Reson. Chem.* **2023**, *61* (8), 473-480. DOI: https://doi.org/10.1002/mrc.5355
(5) Jacob, D.; Deborde, C.; Lefebvre, M.; Maucourt, M.; Moing, A. NMRProcFlow: a graphical and interactive tool dedicated to 1D spectra processing for NMR-based metabolomics. *Metabolomics* **2017**, *13* (4), 36. DOI: https://doi.org/10.1007/s11306-017-1178-y
(6) Pang, Z.; Lu, Y.; Zhou, G.; Hui, F.; Xu, L.; Viau, C.; Spigelman, Aliya F.; MacDonald, Patrick E.; Wishart, David S.; Li, S.; et al. MetaboAnalyst 6.0: towards a unified platform for metabolomics data processing, analysis and interpretation. *Nucleic Acids Res.* **2024**, *52* (W1), W398-W406. DOI: https://www.doi.org/10.1093/nar/gkae253

(7) Xue, J.; Wang, J.; Gong, P.; Wu, M.; Yang, W.; Jiang, S.; Wu, Y.; Jiang, Y.; Zhang, Y.; Yuzyuk, T.; et al. Simultaneous quantification of alpha-aminoadipic semialdehyde, piperideine-6-carboxylate, pipecolic acid and alpha-aminoadipic acid in pyridoxine-dependent epilepsy. *Scientific Reports* **2019**, *9* (1), 11371. DOI: https://doi.org/10.1038/s41598-019-47882-2
(8) Engelke, U. F.; van Outersterp, R. E.; Merx, J.; van Geenen, F. A.; van Rooij, A.; Berden, G.; Huigen, M. C.; Kluijtmans, L. A.; Peters, T. M.; Al-Shekaili, H. H.; et al. Untargeted metabolomics and infrared ion spectroscopy identify biomarkers for pyridoxine-dependent epilepsy. *J. Clin. Invest.* **2021**, *131* (15). DOI: https://www.doi.org/10.1172/jci148272
(9) Sellies, L.; Aspers, R. L. E. G.; Feiters, M. C.; Rutjes, F. P. J. T.; Tessari, M. Parahydrogen Hyperpolarization Allows Direct NMR Detection of α-Amino Acids in Complex (Bio)mixtures. *Angew. Chem. Int. Ed.* **2021**, *60* (52), 26954-26959. DOI: https://doi.org/10.1002/anie.202109588
